# Immune-metabolic crosstalk in HNSCC: mechanisms and therapeutic opportunities

**DOI:** 10.3389/fonc.2025.1553284

**Published:** 2025-05-15

**Authors:** Xiyuan Qin, Hui Wu, Jiaqi Pan, Kai Kang, Yujie Shi, Shoushan Bu

**Affiliations:** Department of Stomatology and Perioperative Medicine, The First Affiliated Hospital of Nanjing Medical University, Nanjing, China

**Keywords:** HNSCC, metabolic reprogramming, immune, tumor microenvironment, tumor

## Abstract

Head and neck squamous cell carcinoma (HNSCC) is a prevalent malignancy, characterized by metabolic reprogramming. This reprogramming creates an acidic and hypoxic environment within tumor cells to adapt to metabolic changes. Experimental data indicate that in HNSCC, the metabolic reprogramming of tumor cells regulates immune cells via metabolites or signaling pathways, thereby promoting cancer progression or immune evasion. This article reviews the metabolic reprogramming in HNSCC, including glucose, fatty acids, amino acids, and nucleotide metabolism. These metabolic pathways play crucial roles in the proliferation, differentiation, and effector functions of immune cells, and influence immunosuppressive checkpoints. Additionally, this review explores the potential relationships between metabolic reprogramming, tumor immunity, and related treatments. Thus, targeting metabolic reprogramming and interactions between immune cells may help overcome therapeutic resistance in HNSCC patients.

## 1 Introduction

Head and neck squamous cell carcinoma (HNSCC) is the most common malignancy in the head and neck region, with 890,000 new cases and 450,000 deaths reported in 2018. The incidence of HNSCC is rising and is projected to increase by 30% by 2030, resulting in approximately 1.08 million new cases annually (1). The etiology of HNSCC is multifactorial, linked to human papillomavirus (HPV), excessive alcohol consumption, tobacco use, diet, and genetics (1–3). Despite various treatments such as surgery, chemotherapy, radiotherapy, and photodynamic therapy, HNSCC remains a highly refractory tumor with a tendency for early metastasis, high recurrence, and low survival rates (4). Moreover, the mechanisms underlying HNSCC development remain unclear.

**Figure 1:**
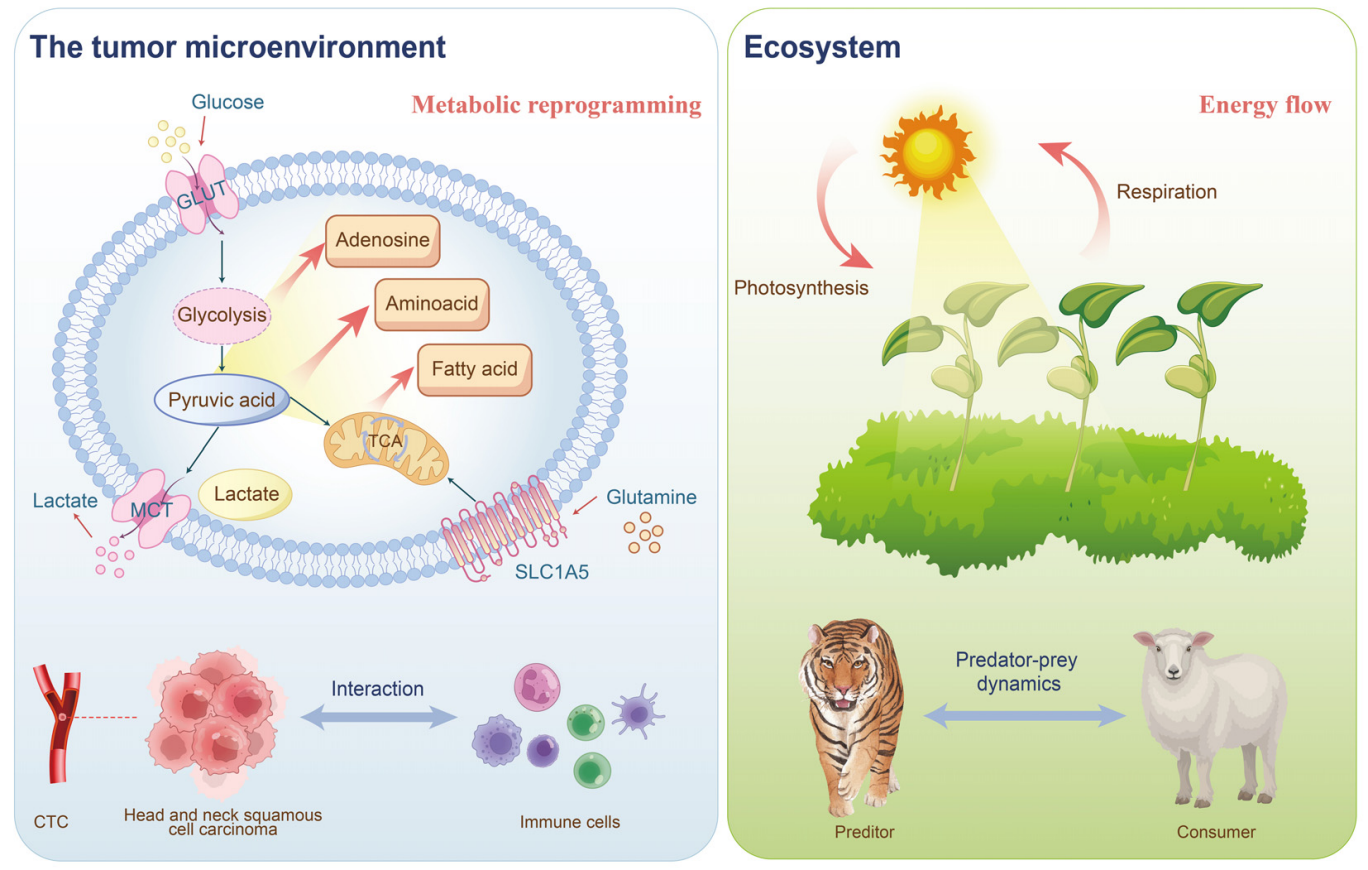
The tumor immune microenvironment of HNSCC can be analogized to an ecosystem: the interactions between tumor cells and immune cells resemble predator-prey and competitive relationships in an ecosystem; CTCs represent spatial heterogeneity akin to species distribution within an ecosystem; and the metabolic reprogramming of tumor cells parallels energy flow dynamics in ecological systems.

Metabolism and immune response are pivotal in cancer research, with metabolic reprogramming and immune escape recognized as cancer hallmarks (5). The metabolic machinery and metabolites directly impact the differentiation and function of immune cells. Therefore, altering these metabolic mechanisms and the production of metabolites can modulate immune function (6). The bioenergetic metabolism of cancer cells, characterized by increased glucose uptake and abundant lactate secretion, along with alterations in the tricarboxylic acid (TCA) cycle, significantly affects the immunological tumor microenvironment (TME) (7). HNSCC serves as an ecological model for studying complex interactions within the TME (**Figure 1**) (8). This environment is highly heterogeneous and includes various cell types, such as tumor cells, immune cells, fibroblasts, and vascular endothelial cells (9), along with complex intercellular interactions and metabolic regulatory networks. These dynamics mirror the interactions between species and the flow of energy within an ecosystem. Similarly, the tumor microenvironment in head and neck cancer comprises diverse cell types, resembling different species in an ecosystem. Interactions among tumor cells, immune cells, and stromal cells parallel the competitive, symbiotic, and predatory relationships observed in natural ecosystems (10). Metabolic reprogramming in HNSCC, such as alterations in glycolysis, amino acid metabolism, and lipid metabolism, resembles the energy flow and resource allocation seen in ecosystems (11–13). Furthermore, the immunosuppressive environment in HNSCC, characterized by phenomena such as T cell exhaustion and an increase in regulatory T cells, can be likened to the predator-prey dynamics in ecosystems (14, 15). Spatial heterogeneity within HNSCC results in varying cell types, metabolic states, and levels of immune infiltration across different regions, akin to the distribution of species and resource variations across different habitats. The resistance mechanisms of HNSCC to radiotherapy, chemotherapy, and immunotherapy can be analogized to species adapting to environmental pressures in ecosystems (16).This new field, termed immunometabolism (17), is explored in terms of how core metabolic alterations in HNSCC influence natural and therapy-driven immunosurveillance, and the potential of targeting such changes to enhance anticancer immune responses.

## 2 Effects of glucose metabolism and its metabolites on immune regulation in HNSCC

### 2.1 The impact of glucose metabolism on immune regulation in HNSCC

**Figure 2:**
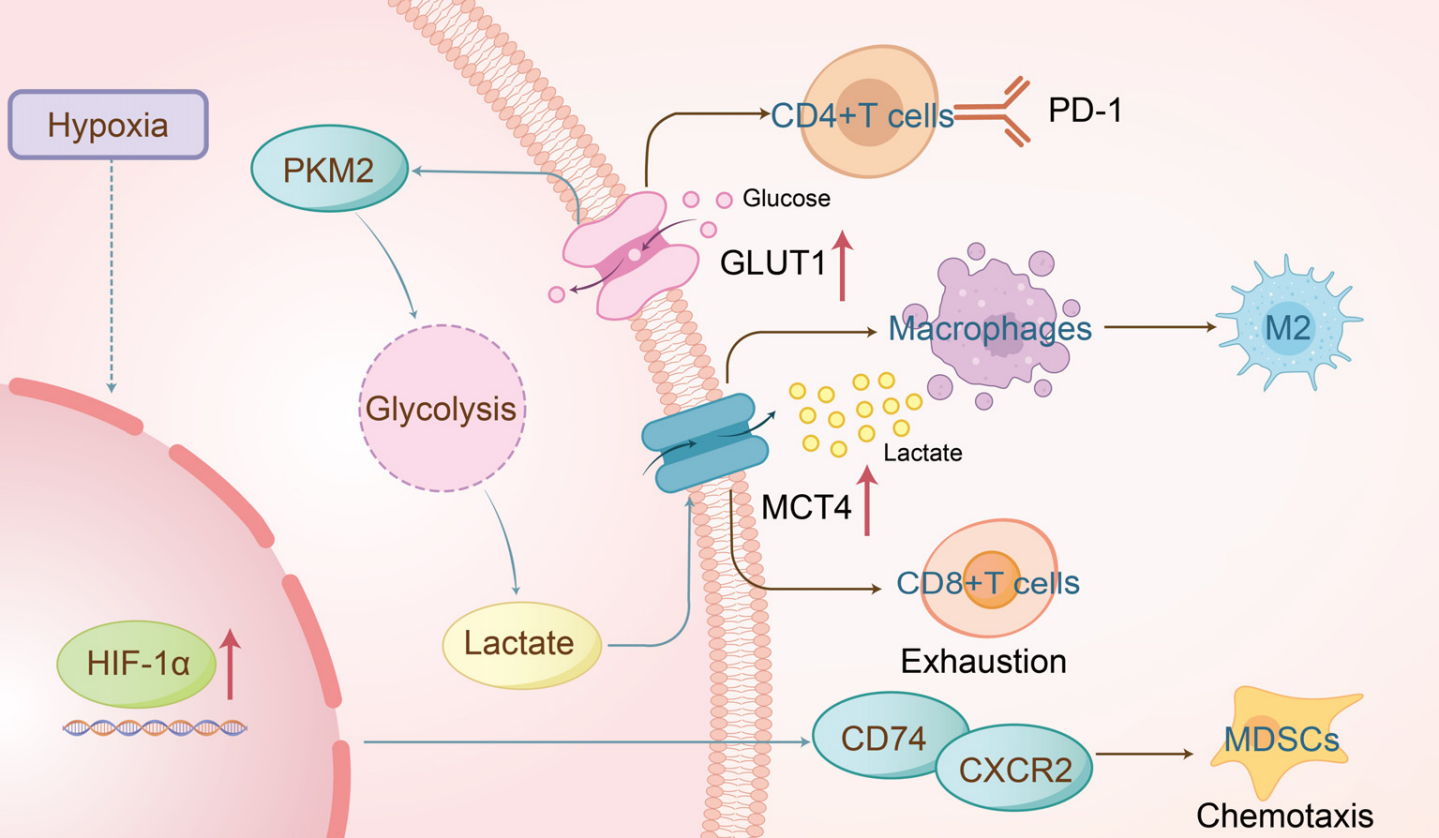
The overexpression of GLUT1 promotes glycolysis in HNSCC, leading to excessive PD-1 expression on CD4^+^ T cells and ultimately resulting in tumor immune escape. Lactate produced during glycolysis is transported extracellularly via MCT4, which drives macrophage polarization toward the pro-tumor M2 phenotype and may also contribute to CD8^+^ T cell exhaustion. Additionally, the hypoxic tumor microenvironment activates HIF-1α, which mediates the formation of the CD74/CXCR2 complex, thereby recruiting immunosuppressive MDSCs to facilitate immune suppression.

In low-oxygen conditions, cells depend on glycolysis, bypassing oxygen-reliant mitochondrial metabolism for energy (18). Unlike normal cells, cancer cells exhibit a preference for glycolysis in the cytoplasm even when oxygen is available, a phenomenon known as the “Warburg effect” or “aerobic glycolysis” (19). This effect is also observed in HNSCC (20). Key regulators of aerobic glycolysis include the “high-affinity” glucose transporter GLUT1, which is a critical rate-limiting factor for cellular glucose uptake and metabolism, lactate exporter monocarboxylate transporter 4 (MCT4), and glycolytic enzymes such as hexokinase 2, phosphofructokinase 1, and enolase 1, along with low-activity pyruvate kinase M2 (PKM2) (21). Research by Christian H. Ottensmeier et al. utilized a mouse model of primary lung epithelial cell tumors (TC-1) overexpressing GLUT1 and assessed extracellular acidification rate (ECAR) as an indicator of glycolysis. An increase in ECAR was noted both in control and GLUT1-transduced TC-1 cells, particularly in those overexpressing GLUT1, demonstrating a negative correlation between high GLUT1 expression and tumor-infiltrating lymphocyte (TIL) infiltration in HNSCC, which delayed tumor regression (**Figure 2**) (22). Similarly, Kun Wu’s research linked lymph node metastasis and recurrence in oral squamous cell carcinoma (OSCC) with increased PD-1 expression and glycolysis in CD4^+^ T cells, indicating potential regulatory mechanisms in OSCC progression (23). Furthermore, Rosemarie Krupar’s studies highlighted the clinical significance of glycolysis-driven immunosuppression in HNSCC through genomic profiling, showing improved survival in patients with favorable immune and metabolic genetic profiles (high CD8A, high mitochondrial-rich COX5B, low GLUT1) (24). These findings illustrate how glycolysis regulates immune cells (25) and immunosuppressive checkpoints in HNSCC, ultimately modulating the tumor’s immune environment.

### 2.2 The role of glycolytic metabolite lactate in immune regulation in HNSCC

Lactate, a principal byproduct of glucose’s glycolytic metabolism, exerts immunosuppressive effects in cancer (26) and plays a significant role in immune regulation in HNSCC. Studies have shown that lactate, produced by upregulated PKM2, promotes tumor progression and galectin-9-mediated immunosuppression through NF-κB signaling in HNSCC, linking metabolic reprogramming with immune regulation (27). Fusobacterium nucleatum, a Gram-negative anaerobic bacterium, has been found to enhance GLUT1 aggregation in the plasma membrane and glycolysis via activation of the GalNAc-autophagy-TBC1D5 signaling pathway, leading to extracellular lactate accumulation and the formation of M2-like tumor-associated macrophages (TAMs) (**Figure 2**). Concurrent inhibition of GalNAc and GLUT1 facilitated the formation of M1 anti-tumor macrophages and regression of OSCC (28). Lactate has also been implicated in immune escape mechanisms in HNSCC. Ruijie Wang et al. demonstrated that IL-11 overexpression promoted tumor progression and CD8^+^ T cell dysfunction *in vivo*; conversely, IL-11 knockout reversed lactate-induced CD8^+^ T cell exhaustion (29). Clinical data further indicate that peripheral lymphocytes and lactate dehydrogenase levels correlate with the response and survival outcomes of immune checkpoint inhibitors in head and neck cancer (30), underscoring lactate’s role in suppressing immune function and facilitating immune evasion by tumors.

### 2.3 Impact of hypoxic metabolic environment on immune regulation in HNSCC

Under hypoxia or pseudohypoxia (where oxygen is present but cannot be properly utilized due to alterations in oxygen-sensing pathways), cells activate numerous adaptive responses. These responses are coordinated by various cellular pathways, most of which are controlled by a common factor, namely the hypoxia-inducible factor (HIF) (31). Hypoxic microenvironments are prevalent in most solid tumors, with research showing that cancer cells adapt to hypoxia through signaling pathway alterations. Hypoxia induces immune tolerance (32), weakening cytotoxic T cell function and promoting regulatory T cell recruitment, thereby diminishing tumor immunogenicity (33). Quynh-Thu Le et al. identified Galectin-1 as a hypoxia-regulated protein and prognostic marker in HNSCC, noting a strong negative correlation between Galectin-1 and CD3 staining (34). Furthermore, Dan P. Zandberg et al. reported that increased oxidative metabolism in tumor cells, during PD-1 resistance, exacerbated intratumoral hypoxia and reduced CD8^+^ T cell infiltration. Hypoxia-inducible factors (HIFs), particularly HIF-1α and HIF-2α, play central roles in modulating immune evasion (33) by regulating the chemotaxis of CD11b^+^Gr^-^1^+^ myeloid cells through binding to CD74/CXCR2 and CD74/CXCR4 complexes(**Figure 2**) (35), activating the p38/MAPK and PI3K/AKT signaling pathways (36). These findings suggest a significant role for hypoxia in regulating the immune microenvironment of HNSCC, warranting further exploration of its mechanisms.

## 3 Effects of other metabolites and metabolic pathways on immune regulation in HNSCC

### 3.1 Amino acid’s role in immune regulation in HNSCC

**Figure 3:**
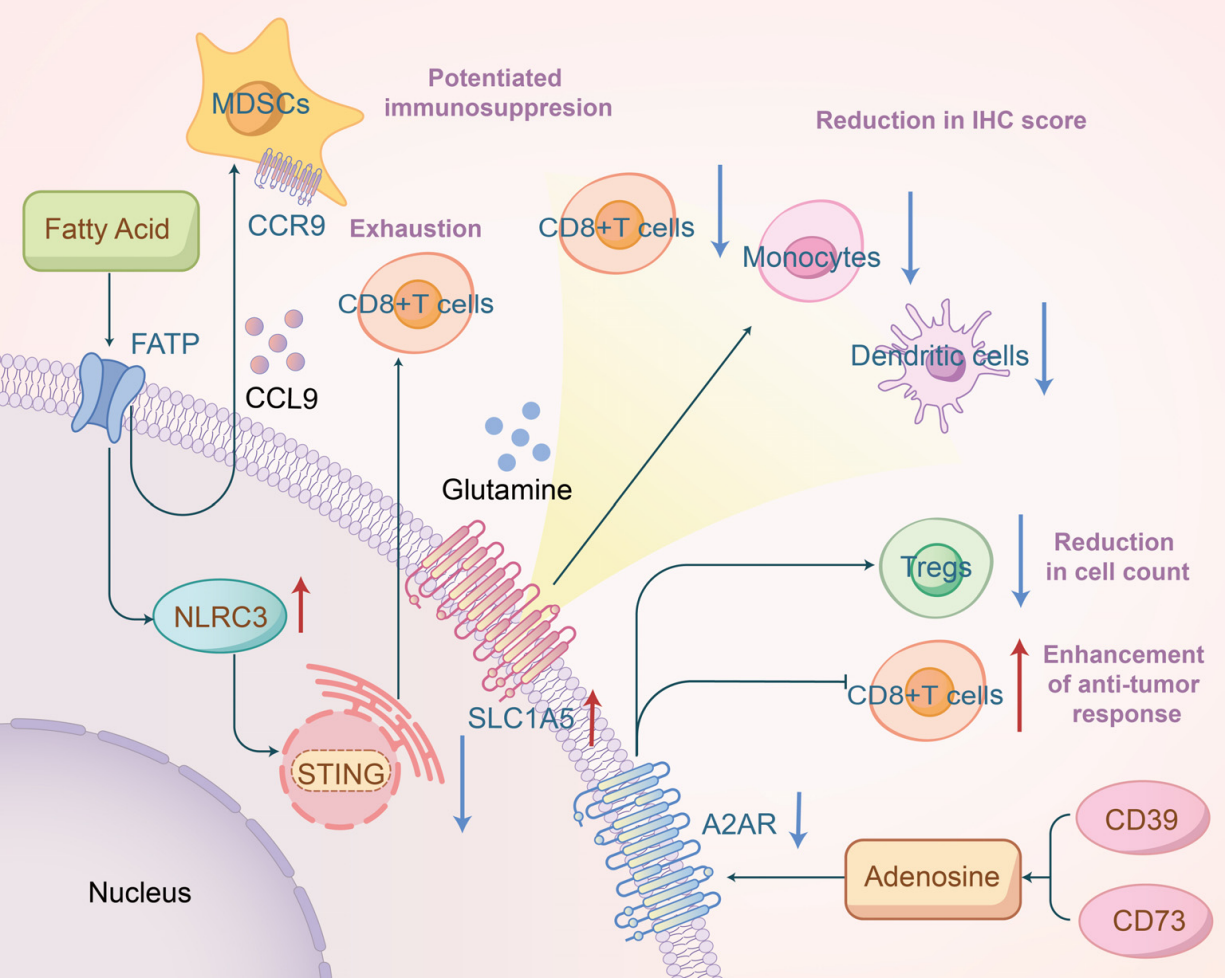
Fatty acids promote NLRC3 expression to inhibit the STING pathway, thereby inducing exhaustion of CD8^+^ T cells, and mediate CCL9/CCR9 to enhance the immunosuppressive function of MDSCs; overexpression of SLC1A5 is associated with reduced immunohistochemical scores of related immune cells; inhibition of A2AR may decrease the number of Treg cells and promote antitumor immune responses of CD8^+^ T cells.

Cancer’s progression from a localized tumor to widespread metastatic disease is complex and multifaceted, making it a leading cause of death among cancer patients (37). The primary agents of this process are circulating tumor cells (CTCs), which detach from the primary tumor and disseminate through the bloodstream to colonize other organs (38). Indoleamine 2,3-dioxygenase 1 (IDO1) is an enzyme involved in the catabolism of the essential amino acid L-tryptophan; it depletes this amino acid and contributes to immune suppression and tolerance in the tumor microenvironment. Studies indicate that in patients with locally advanced head and neck cancer undergoing chemoradiation, IDO1 acts as a surrogate biomarker for an “inflamed” good prognosis phenotype at baseline. Conversely, persistent overexpression of IDO1 at treatment’s end may negate the effects of immunogenic cell death induced by chemoradiation (39).Glutamine metabolism is vital for various cellular functions, including nucleotide synthesis, amino acid production, redox balance, glycosylation, extracellular matrix production, autophagy, and epigenetics (40). It is extensively utilized by macrophages and neutrophils, and glutamine availability also governs the production and secretion of pro-inflammatory cytokines (IL-6, IL-1, and TNF) by macrophages (41). Glutamine is critical for immune regulation in HNSCC. Ying-Chieh Liu, through database analysis, found that higher expression of SLC1A5 (amino acid transporters) in tumors was associated with significantly lower immune scores in CD8, monocytes, and dendritic cells, and higher scores in M0 and M1 macrophages (**Figure 3**). Disruptions in immune modulation, metabolism, and oxidative stress were linked to SLC1A5 aberrations in HNSCC (42). Meanwhile, An Song reported that glutamine levels in HNSCC improved post-radiation therapy, correlating with upregulated expression of the glutamine transporter SLC1A5. This study also demonstrated that inhibiting glutamine metabolism in conjunction with radiotherapy enhanced the expression of CD47, an immune checkpoint receptor that protects cells from macrophage phagocytosis, thereby hindering phagocytosis and reducing treatment efficacy (43). This indicates that targeting glutamine metabolism might offer a novel approach to cancer therapy.

### 3.2 The impact of fatty acid metabolism on immune regulation in HNSCC

Multiple studies have demonstrated that aberrant signaling pathways or nutrient competition in the tumor microenvironment can induce phenotypic reprogramming of fatty acid metabolism and alter the function of tumor-infiltrating immune cells, thereby impacting the efficacy of cancer immunotherapy (44). Blake R. Heath et al. suggested that obesity might create an interferon-I-deprived tumor microenvironment, leading to the expansion of inhibitory myeloid cell clusters and a reduction in effector T cells. This process may involve saturated fatty acids inducing NLRC3 expression, a DNA-binding protein that inhibits the STING pathway, effectively suppressing the STING-IFN-I pathway in HNSCC cells, leading to T cell exhaustion and reduced HNSCC immunogenicity (45). Consistently, other researchers have shown that a high-fat diet-induced obesity significantly promotes OSCC development and alters the local immune microenvironment by expanding CD11b^+^Gr1^+^ myeloid-derived suppressor cells (MDSCs), potentially through recruitment via the CCL9/CCR1 axis and enhancing MDSC immunosuppressive functions via intracellular fatty acid uptake(**Figure 3**) (46). Marwah M. Albakri et al. also discovered that fatty acids secreted by HNSCC induced M2-like macrophage formation (47). These findings indicate that in HNSCC, the aberrant accumulation of lipid metabolites (e.g., short-chain fatty acid, long chain fatty acid, cholesterol, etc.) in tumor-infiltrating myeloid cells, including MDSCs, DCs, and TAMs, skews these immune cells towards immunosuppressive and anti-inflammatory phenotypes through metabolic reprogramming. Exploring ways to overcome fatty acid metabolic dysregulation could improve immunotherapy outcomes for HNSCC.

### 3.3 The role of adenosine signaling pathway in immune regulation in HNSCC

Adenosine is an evolutionarily conserved metabolic regulator that links energy status to physiological processes, including immune regulation and cell proliferation. Tumors create an adenosine-rich immunosuppressive microenvironment by increasing ATP release in dying and stressed cells and converting it to adenosine via extracellular enzymes (48). The accumulation of nucleoside adenosine in the tumor microenvironment suppresses various immune cells’ anti-tumor functions, including cytotoxic T cells and natural killer cells, by binding to adenosine A2A receptors (A2ARs) (5, 49). In HNSCC, the adenosine signaling pathway plays an indispensable role in immune surveillance. Recent studies have highlighted that A2AR expression is significantly correlated with HIF-1α, CD73, CD8, and Foxp3, and that blocking A2AR significantly reduces the number of CD4^+^Foxp3^+^ Tregs while enhancing CD8^+^ T cell anti-tumor responses(**Figure 3**) (50). Additionally, some studies have revealed that co-inhibition of the adenosine 2B receptor and programmed death ligand 1 promotes the recruitment and cytotoxicity of natural killer cells in OSCC (51). Magis Mandapathil et al. demonstrated that adenosine deaminase (ADA), responsible for deaminating immunosuppressive adenosine to inosine, increases CD4^+^ effector T cells’ sensitivity to inhibitory signals transmitted by adenosine receptors in HNSCC patients, leading to extracellular adenosine accumulation and affecting the tumor microenvironment (52). Ectonucleotidases CD39 (also known as NTPDase 1) and CD73 (5’-NT) are cell surface molecules that play an indispensable role (49). Targeting CD39 and CD73 activity to inhibit adenosine production is a promising strategy to enhance anti-tumor immunity.

## 4 Regulation of immune metabolism in HNSCC by related signaling pathways

### 4.1 Immune regulation by the PI3K−Akt−mTOR pathway in HNSCC

**Figure 4:**
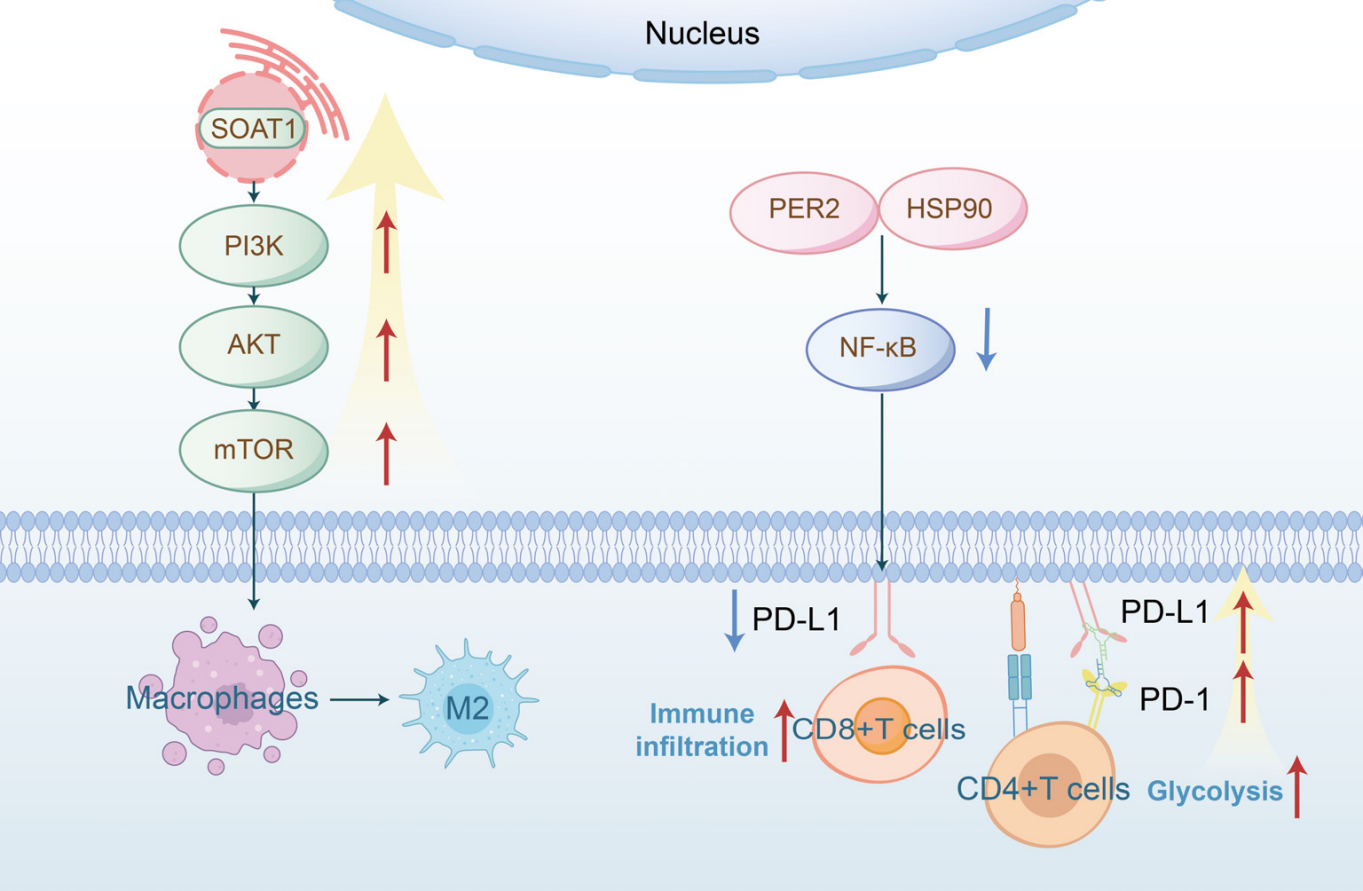
SOAT1 (a lipid metabolism enzyme) activates the PI3K−Akt−mTOR signaling pathway, thereby promoting macrophage polarization toward the M2 phenotype; PER2 binds to HSP90 and inhibits the NF-κB signaling pathway, leading to decreased PD-L1 expression and thereby enhancing CD8^+^ T cell immune infiltration; overexpression of the PD-L1/PD-1 axis may increase glycolysis in CD4^+^ T cells, ultimately leading to lymph node metastasis.

The PI3K-mTOR pathway, frequently activated in cancer, controls cell growth and metabolism. mTOR signaling regulates amino acid, glucose, nucleotide, fatty acid and lipid metabolism (53). Recent studies have identified important regulatory roles of mTOR in the differentiation, activation, and functional properties of immune cells, where mTOR’s function is to coordinate and shape immune effector responses (54, 55). The Akt-mTOR pathway is known to be activated in HNSCC (56). Numerous studies focus on the regulation of immunity by the mTOR signaling pathway through metabolic reprogramming in HNSCC. Tyrosine phosphorylation of HER3 (a member of the ErbB protein family) and PI3K were identified as the basis for abnormal PI3K/AKT/mTOR signaling in PIK3CA wild-type HNSCC. It has been discovered that HER3 blockade inhibited HER3-PI3K-AKT-mTOR oncogenic signaling and simultaneously reversed the immunosuppressive tumor microenvironment, demonstrating that co-targeting HER3 and PD-1 led to tumor growth inhibition and subsequently enhanced therapeutic immune responses (57). There is convincing evidence that PD-L1 promotes HNSCC cell growth through mTOR signaling, further supporting the role of mTOR metabolic signaling in immune evasion in HNSCC (58). Additional research has discovered that SOAT1, a key enzyme in lipid metabolism, activated the PI3K/AKT/mTOR pathway and promoted M2-like polarization of TAMs, thus promoting OSCC growth both *in vitro* and *in vivo*(**Figure 4**) (59). The mTOR signaling pathway is also implicated in regulating the immune system through its effects on glucose metabolism. Akt, by interacting with multiple downstream targets such as the 160 KDA Akt substrate (AS160), plays a crucial role. AS160 is known to inhibit the translocation of GLUT4 to the membrane, a vital glucose transporter crucial for cellular energy needs. Activation of Akt diminishes the inhibitory effect of AS160, leading to increased activity of GLUT4. Consequently, this activation elevates glucose absorption in cancerous cells, thereby augmenting glycolytic flux (60). Ellen C. Moore et al. has discovered that the concomitant use of rapamycin, an inhibitor of mTOR, with anti-PD-L1 therapy, significantly boosts antigen-specific CD8^+^ T cell responses and facilitates the infiltration of immune cells (61). It is imperative for subsequent research to delineate more precisely the roles of the mTOR pathway in both tumor and immune cells within the context of HNSCC.

### 4.2 Regulation of immune metabolism in HNSCC by the NF-κB pathway

The NF-κB transcription factor is crucial in various normal cellular processes, such as inflammation and cell survival, and plays a role in the molecular pathogenesis of cancer (62). As a transcription factor for anti-apoptotic genes, NF-κB promotes tumor survival in various cancers, including HNSCC. Matrix metalloproteinase 9 (MMP-9), associated with lymph node metastasis and reduced survival rates, is synergistically upregulated by pro-inflammatory cytokines and growth factors in an NF-κB-dependent manner (63, 64). Research indicates that under glucose-deficient conditions, both CXCL8 mRNA and its protein IL-8 are elevated in cancer cells in an NF-κB-dependent manner. Furthermore, targeting CXCL8 signaling enhances the sensitivity of HNSCC to anlotinib by reducing tumor-associated macrophage-derived agrin (65). The NF-κB pathway is not only involved in the regulation of glucose metabolism but also plays a role in lipid metabolism. Similarly, studies have demonstrated that PER2 (Period circadian regulator 2) binds to HSP90, thereby inhibiting the IKK/NF-κB pathway and downregulating PD-L1 expression, which enhances the cytotoxic activity of CD8^+^ T cells against OSCC (**Figure 4**) (66).

### 4.3 Regulation of metabolic reprogramming in HNSCC by the PD-1\PD-L1 signaling pathway

Malignant tumor cells evade anti-tumor immune responses by promoting negative signaling pathways such as PD-1/PD-L1 (67). Specifically, PD-1 upregulation inhibits the effector functions and expansion of T cells within the tumor microenvironment, thus enabling tumor cells to escape immune surveillance (68). Literature indicates that elevated levels of PD-1 and glycolysis in CD4+ T cells are positively correlated with lymph node metastasis in OSCC (**Figure 4**) (23). Sphingosine kinases (SPHKs), crucial rate-limiting enzymes, exist as two subtypes, SPHK1 and SPHK2, which catalyze the phosphorylation of sphingosine (SPH) to sphingosine-1-phosphate (S1P). Research by Qi Fang et al. shows that SPHK1 promotes immune escape in HNSCC by regulating the MMP1-PD-L1 axis. The combination of PD-1 immunotherapy and targeted metabolic reprogramming has become a critical research focus in cancer therapeutics, offering synergistic effects that may overcome the limitations of single-agent therapies, enhance antitumor efficacy, and address drug resistance.

## 5 Therapeutic prospects of targeting immune metabolism in HNSCC

Surgical resection remains the primary treatment for HNSS, with chemotherapy and radiotherapy serving as key adjuvant therapies. The studies mentioned above provide a deeper understanding of metabolic interventions in HNSCC and immune cells, which may reveal novel therapeutic targets. Immunometabolic pathways in HNSCC hold significant potential for therapeutic development (**Table 1**). Glycolytic metabolism may contribute to the suboptimal efficacy of immunotherapy in HNSCC. To address this, Yong Teng et al. used ganetespib, an inhibitor of heat shock protein 90 (HSP90), as a pharmacological model. Their prior research showed that HSP90 inhibition suppresses glycolytic flux in HNSCC cells by downregulating PKM2 at both the transcriptional and post-translational levels. Further studies revealed that combining ganetespib with radiotherapy attenuates radiation-induced PKM2 upregulation and enhances T cell-mediated antitumor immunity, resulting in superior antitumor efficacy compared to either treatment alone (69). Lactic acid is also a viable immune-metabolic target. Sun M Lim et al. found that pembrolizumab-based combination therapy reduced the expression of lactate-producing genes (including SLC16A3 and LDHA) in the EGFR ^HIGH^ MET ^HIGH^ subpopulation of HNSCC. This study highlighted the remodeling of the TME by the combination therapy, providing a theoretical basis for additional therapeutic strategies combining amivantamab (a bispecific EGFR-MET antibody) with PD-1 immunotherapy (70). Extensive tumor necrosis and localized hypoxia in HNSCC may contribute to poor chemotherapeutic response or even drug resistance. To address this, Tong Wu et al. developed a hypoxia-adaptive nanocomposite TiO_2_@Ru@siRNA for the prevention and treatment of OSCC. Under visible light excitation, this material efficiently induces HIF-1α gene silencing and OSCC cell eradication while remodeling the immunosuppressive microenvironment by downregulating key immunosuppressive factors and activating T-cell-mediated antitumor immunity (71). Regarding the previously discussed glutamine and adenosine metabolic reprogramming, related drugs have already entered clinical trials. In HNSCC, mutations in Nuclear factor erythroid 2-related factor 2 (Nrf2) increase intratumoral recruitment of polymorphonuclear myeloid-derived suppressor cells (PMN-MDSCs) and reduce M1 macrophages, inducing radioresistance. Li Guan et al. (72) discovered that the glutaminase inhibitor CB-839 can reverse these changes. Zhi-Jun Sun et al. demonstrated that blocking with SCH58261 (an A2AR antagonist) significantly reduced the population of CD4^+^Foxp3^+^ Treg cells and enhanced the anti-tumor response of CD8^+^ T cells (50). Weiwei Deng ‘s team also discovered that blocking adenosine with MEDI9447 (a CD73-specific monoclonal antibody) can alter the exhausted phenotype of T cells (73). Research on drugs targeting fatty acid metabolic reprogramming remains limited and is currently in the experimental stage. Marwah M Albakri et al. found that fatty acids in HNSCC promote macrophage polarization toward the M2 phenotype, and the fatty acid oxidation inhibitor Etomoxir can reverse this polarization (47). This drug shows promising clinical potential.

**Table 1:** Drugs targeting immunometabolism in HNSCC.

Drugs name	Action target	Targeted metabolism	Targeted immune	Clinical trial
Ganetespib	HSP90	Suppresses tumor glycolytic flux	facilitate tumor infiltration of cytotoxic T cells	NCT02334319
Amivantamab	EGFR and MET	increased expression of genes implicated in production of lactate(SLC16A3)	enhanced infiltration of granzyme B–producing CD8 T cells	NCT05908734
TiO_2_@Ru@siRNA	HIF-1α	hypoxia relief	activation of CD4 and CD8 T lymphocytes	Preclinical phase
CB-839	GLS1	Inhibit the conversion of glutamine to glutamate	Reduce the expression of PMN-MDSC-attracting chemokines (including CXCL1, CXCL3, and CSF3).	NCT03528642
SCH58261	A2AR	inhibit adenosine-A2AR interaction	reduced the population of CD4^+^ Foxp3^+^ Tregs and enhanced the anti-tumor response of CD8^+^ T cells	Preclinical phase
anti-CD73 monoclonal antibody (mAb)	CD73	inhibit adenosine generation	reverse the ‘exhausted’ phenotype of CD4^+^ and CD8^+^ T cells	NCT02503774
Etomoxir	CPT1	Inhibition of fatty acid oxidation	Reverse M2 macrophage polarization	Preclinical phase
Rapamycin	mTOR	attenuate tumor lactate production	Enhances Immune-Mediated Tumor Clearance	NCT01195922
reparixin	CXCL1/2	Hypoxia-driven glucose deprivation triggers CXCL8 upregulation via HIF-1α/NF-κB axis	Inhibit CLU synthesis in tumor-associated TAMs	Preclinical phase
IDOi (BMS986205)+ nivolumab	IDO1and PD-1	Modulate tryptophan metabolism	Block the binding of PD-1 to PD-L1	NCT03854032

Targeting relevant signaling pathways that regulate immunometabolism in HNSCC presents promising application prospects for improving therapeutic efficacy. Clinical trials are currently evaluating mTOR inhibitors in combination with various treatment modalities for HNSCC (74). John H. Lee et al. found that rapamycin reduces lactate levels in HNSCC tumors, thereby alleviating the inhibitory effect of lactate on perforin release by CD8^+^ T cells (75). The research group led by Xu Wang found that IL-8 supplementation stimulates TAMs to synthesize clusterin (CLU), which counteracts oxidative stress in HNSCC cells under glucose-deficient conditions. Additionally, in two xenograft models, pharmacological blockade of CXCL8 signaling (using reparixin) sensitized HNSCC cells to nutrient deprivation therapy (anlotinib) (65). Although immune checkpoint blockade therapy has advanced rapidly in recent years, a subset of patients still fail to achieve satisfactory treatment outcomes. Eric V. Mastrolonardo and colleagues conducted a clinical trial demonstrating that combining the IDO1 inhibitor BMS-986205 with the PD-1 inhibitor Nivolumab enhanced T cell activity and boosted immune-mediated antitumor responses in treatment-naïve, surgically resectable HNSCC patients (76).

## 6 Conclusion

Previous studies have demonstrated that HNSCC acquires immune evasion capabilities by altering metabolic pathways. Targeting these pathways, along with anti-immune evasion treatments, holds significant clinical potential. However, the specific mechanisms require further detailed investigation. Discussion of mitochondrial function in regulating immunity remains insufficient and demands more comprehensive explanation.
